# Comprehensive Genetic Characterization of *Salmonella enterica* Serovar Gallinarum Biovar Gallinarum from the Poultry Industry in South Korea

**DOI:** 10.3390/ani16213404

**Published:** 2026-10-28

**Authors:** Min Beom Kim, O-Mi Lee, So-Hee Lee, Hye-Rin Lee, Yeong Ju Yu, Sung-Il Kang, Moon Her, Jin Hyun Kim

**Affiliations:** Avian Disease Division, Animal and Plant Quarantine Agency, 177 Hyeoksin 8-ro, Gimcheon-si 39660, Gyeongsangbuk-do, Republic of Korea; minbum5935@gmail.com (M.B.K.); lomi78@korea.kr (O.-M.L.); tdj04051@gmail.com (S.-H.L.); leehr4612@gmail.com (H.-R.L.); yeongju9213@gmail.com (Y.J.Y.); ksilion@korea.kr (S.-I.K.); herm@korea.kr (M.H.)

**Keywords:** *Salmonella enterica* subsp. *enterica* serovar Gallinarum biovar Gallinarum, fowl typhoid, whole-genome sequencing, genomic characterization

## Abstract

*Salmonella enterica* subsp. *enterica* serovar Gallinarum biovar Gallinarum (SG) causes fowl typhoid, an important poultry disease, but the genetic diversity and phylogenetic relationships among Korean SG isolates remain insufficiently investigated. We analyzed 26 Korean SG isolates collected between 2002 and 2026 and compared them with 57 publicly available SG genomes from other countries using whole-genome sequencing. Using a cutoff of 100 core-genome single-nucleotide polymorphisms, 83 isolates were classified into eight clusters and ten singletons, with all Korean isolates assigned to the same cluster. Five sequence types (ST78, ST331, ST13540, ST14504, ST14529) were identified. ST78 was predominant, whereas ST14504 and ST14529 were identified exclusively among Korean isolates. Bayesian phylogenetic analysis estimated the time to the most recent common ancestor of the Korean isolates to be approximately 1990. Genomic analyses revealed differences in *Salmonella* pathogenicity islands, virulence genes, plasmid replicons, antimicrobial resistance genes, and quinolone resistance-determining region mutations. A resistance profile comprising *aac(3)-VIa*, *aadA1*, *sul1*, and *qacEΔ1* was observed exclusively in 12 Korean isolates; of these, eleven harbored only the IncFII(S) plasmid replicon, and IncX1 was also detected in one isolate, together with *bla*_TEM-1B_ and *tet(A)*. These findings provide valuable genomic and epidemiological insights into SG and may contribute to improved control of fowl typhoid.

## Simple Summary

Fowl typhoid (FT) is an important bacterial disease in poultry caused by *Salmonella enterica* subsp. *enterica* serovar Gallinarum biovar Gallinarum (SG) that has led to substantial economic losses in the poultry industry. Although FT continues to occur in South Korea, the genetic characteristics and population structure in South Korea remain poorly understood. In this study, we analyzed 26 SG isolates collected from poultry in South Korea between 2002 and 2026 and compared them with 57 isolates from other countries using whole-genome sequencing. The Korean isolates formed a genetically related group that was distinct from isolates from other geographical regions, while also exhibiting genetic diversity within the Korean population. Several sequence types and antimicrobial and disinfectant resistance profiles were identified exclusively among the Korean isolates. Our findings provide a better understanding of the genetic diversity and evolution of SG in South Korea and may contribute to improved genomic surveillance and control strategies for FT in poultry.

## 1. Introduction

*Salmonella enterica* subsp. *enterica* serovar Gallinarum biovar Gallinarum (SG) is a host-restricted bacterial pathogen that causes fowl typhoid (FT), an acute or chronic septicemic disease primarily affecting chickens [1]. FT is associated with high morbidity and mortality in affected poultry flocks and causes substantial economic losses in the poultry industry [1,2]. The incidence of FT has declined markedly in many developed countries following the implementation of coordinated control measures, including systematic surveillance, culling of infected flocks, improved hygiene, strict biosecurity, and vaccination [3]. Nevertheless, FT remains prevalent in many regions, particularly in developing countries, and continues to pose a significant threat to poultry health and production [4].

In South Korea, FT has been recognized as an important bacterial disease affecting the poultry industry since it was first reported in commercial chickens in 1992 [5]. Following the introduction of a vaccination program using a live attenuated strain (SG9R) in 2001, the incidence of FT declined substantially; however, outbreaks have continued to occur annually despite ongoing vaccination, surveillance, and improvements in farm management and biosecurity [5,6,7]. Furthermore, the use of antimicrobials to treat and control bacterial infections in poultry has raised concerns regarding the emergence and persistence of antimicrobial-resistant SG [7,8]. The continued occurrence of FT and challenges in disease control, including antimicrobial resistance (AMR), highlight the need for a more comprehensive understanding of the genetic and epidemiological characteristics of SG in South Korea.

Whole-genome sequencing (WGS) provides high-resolution genomic information for discriminating closely related bacterial strains and investigating their genetic relationships, while enabling the simultaneous characterization of diverse genomic features, including AMR and virulence determinants [9]. Previous studies have reported relatively low genetic heterogeneity among SG isolates, with closely related isolates exhibiting highly similar profiles using conventional molecular typing approaches, including pulsed-field gel electrophoresis and multiple-locus variable-number tandem repeat analysis [5,10,11]. Accordingly, WGS-based genomic analyses have been applied to SG isolates collected across different geographical regions and time periods to investigate their genetic diversity, phylogenetic relationships, and other genomic characteristics [12,13,14,15]. However, studies in South Korea have largely focused on the genomic characterization of individual isolates, and the genetic diversity and phylogenetic relationships among Korean SG isolates remain insufficiently investigated [16,17]. Therefore, this study aimed to comprehensively characterize SG isolates from poultry in South Korea using WGS, with a focus on their genomic diversity, epidemiological relationships, AMR, and virulence.

## 2. Materials and Methods

### 2.1. Bacterial Isolation and Identification

A total of 26 SG isolates available for WGS were selected from a previously described collection maintained by the Avian Disease Division of the Animal and Plant Quarantine Agency, Republic of Korea [18], which comprised isolates collected between 2002 and 2026 through routine diagnostic testing of chickens or confirmatory testing for FT (Table S1). The isolates were obtained from Baeksemi, breeders, broilers, and layers. Briefly, suspected SG isolates were obtained from two sources: isolates recovered by the Avian Disease Division of the Animal and Plant Quarantine Agency from organs or tissues collected for diagnostic testing and isolates submitted by veterinary service laboratories in each city and province for confirmatory identification.

The isolates were cultured on MacConkey agar (Difco, Sparks, MD, USA). Presumptive colonies were identified by biochemical testing using the Vitek 2 system (bioMérieux, Lyon, France). Confirmed colonies were serotyped by agglutination tests using O and H antisera (Difco) according to the Kauffmann–White scheme [19]. Because SG and *Salmonella Pullorum* cannot be distinguished by conventional serotyping, the biovars were differentiated using a previously described polymerase chain reaction (PCR) method. This method was also used to distinguish SG9R-derived isolates from field SG isolates [20].

### 2.2. Whole-Genome Sequencing (WGS)

Genomic DNA was extracted from the 26 SG isolates using the DNeasy^®^ Blood & Tissue Kit (QIAGEN, Hilden, Germany). DNA quantity and purity were assessed using a Qubit™ 4 Fluorometer (Thermo, Waltham, MA, USA) and NanoDrop™ 2000 spectrophotometer (Thermo), respectively. Sequencing libraries were prepared using the Illumina^®^ DNA Prep Kit (Illumina, San Diego, CA, USA), followed by paired-end sequencing (2 × 300 bp) on an Illumina MiSeq Platform using the MiSeq Reagent Kit v3 (Illumina).

### 2.3. Assembly of WGS Data and Datasets

Raw sequencing reads for the 26 SG isolates generated in this study were combined with an additional 57 raw sequencing datasets retrieved from the National Center for Biotechnology Information (NCBI) Sequence Read Archive (SRA) database. All raw sequencing reads were processed using fastp v0.24.0 to trim adapter sequences and low-quality bases [21] and assembled de novo using SPAdes v4.3.0 [22]. Assembly quality was assessed using QUAST v5.3.0 according to the criteria for *Salmonella* in EnteroBase: assembly size between 4.0 and 5.8 Mbp, N50 value > 20 kb, number of contigs < 600, and proportion of scaffolding placeholders (N’s) < 3% [23]. To further confirm differentiation between biovar Gallinarum and Pullorum, the *torT* and *I137_14430* genes were examined [24]. Finally, a total of 83 SG isolates, comprising 26 isolates from Korea and 57 isolates from other countries, were included in the final analysis (Table S1).

### 2.4. In Silico Genomic Characterization and Antimicrobial Susceptibility

The assembled genomes were characterized using PubMLST for sequence types (STs) [25], Virulence Factor Database (VFDB) for virulence factors [26], PlasmidFinder for plasmid replicons [27], and ResFinder for AMR genes, chromosomal point mutations, and disinfectant resistance genes [28]. *Salmonella* pathogenicity islands (SPIs) were identified by the Basic Local Alignment Search Tool for nucleotides against reference sequences from the SPIFinder database, using minimum thresholds of 95% for identity and 60% for coverage [29]. Detailed genomic characterization data for 83 SG isolates are provided in Table S2. Antimicrobial susceptibility data for the 26 Korean SG isolates, including minimum inhibitory concentration (MIC) values, were obtained from a previous study [18].

### 2.5. Phylogenetic and Time-Scaled Bayesian Analyses

For phylogenetic and Bayesian analyses, single-nucleotide polymorphisms (SNPs) were identified using Snippy v4.6.0 by mapping the fastp-filtered sequencing reads to the SG strain 287/91 reference genome (accession No. AM933173.1), and a core SNP alignment was generated using Snippy-core [30].

A maximum-likelihood phylogenetic tree was constructed using IQ-TREE with the ModelFinder Plus option and ascertainment bias correction (MFP + ASC), 1000 ultrafast bootstrap replicates, and 1000 SH-aLRT replicates [31]. Pairwise SNP distances were calculated using snp-dists v1.2.0 [32], followed by single-linkage hierarchical clustering using ReporTree v2.6.1 using a 100-SNP threshold [33].

A time-scaled Bayesian analysis was performed using BEAUti v2.7.8 and BEAST v2.7.7 [34]. The Bayesian analysis was calibrated using the collection years of the isolates (1972–2026) as tip dates, with a Uniform(0, ∞) prior specified for the substitution rate. The analysis was configured with the GTR substitution model and four gamma categories, and four combinations of two clock models (strict and lognormal relaxed) and two coalescent population models (constant size and Bayesian skyline) were evaluated. Among the four evaluated model combinations, the strict-clock and constant-size model was selected for the final analysis, with effective sample size (ESS) values ranging from 6572 to 9001, as assessed using Tracer [35]. The final Markov chain Monte Carlo analysis was run for 500 million generations, with samples collected every 50,000 generations. A maximum clade credibility tree was generated using TreeAnnotator with a 10% burn-in. iTOL v7 and FigTree v1.4.4 were used to visualize these analyses and annotate the maximum-likelihood phylogenetic tree [35,36].

### 2.6. Data Availability

The WGS data generated in this study have been deposited in the NCBI SRA under BioProject accession number PRJNA1533643 and are publicly available under BioSample accession numbers SAMN63375196–SAMN63375221.

## 3. Results

### 3.1. WGS Data

The SG WGS dataset comprised 83 genomes, with detailed metadata provided in Table S1. The isolates originated from 14 countries across five continents (Africa, Asia, Europe, North America, and South America) and were obtained from poultry and other birds, with one isolate each from a human and an equine. Host information was unavailable for nine isolates. The 83 genome assemblies exhibited a median size of 4,711,059 bp (range, 4,505,266–4,883,831 bp), a median of 41 contigs (range, 23–182), and a median GC content of 52.2% (range, 52.1–52.3%). Following quality assessment, a reliable genome dataset was obtained for subsequent genomic analyses.

### 3.2. Phylogenetic Relationships and STs

**Figure 1:**
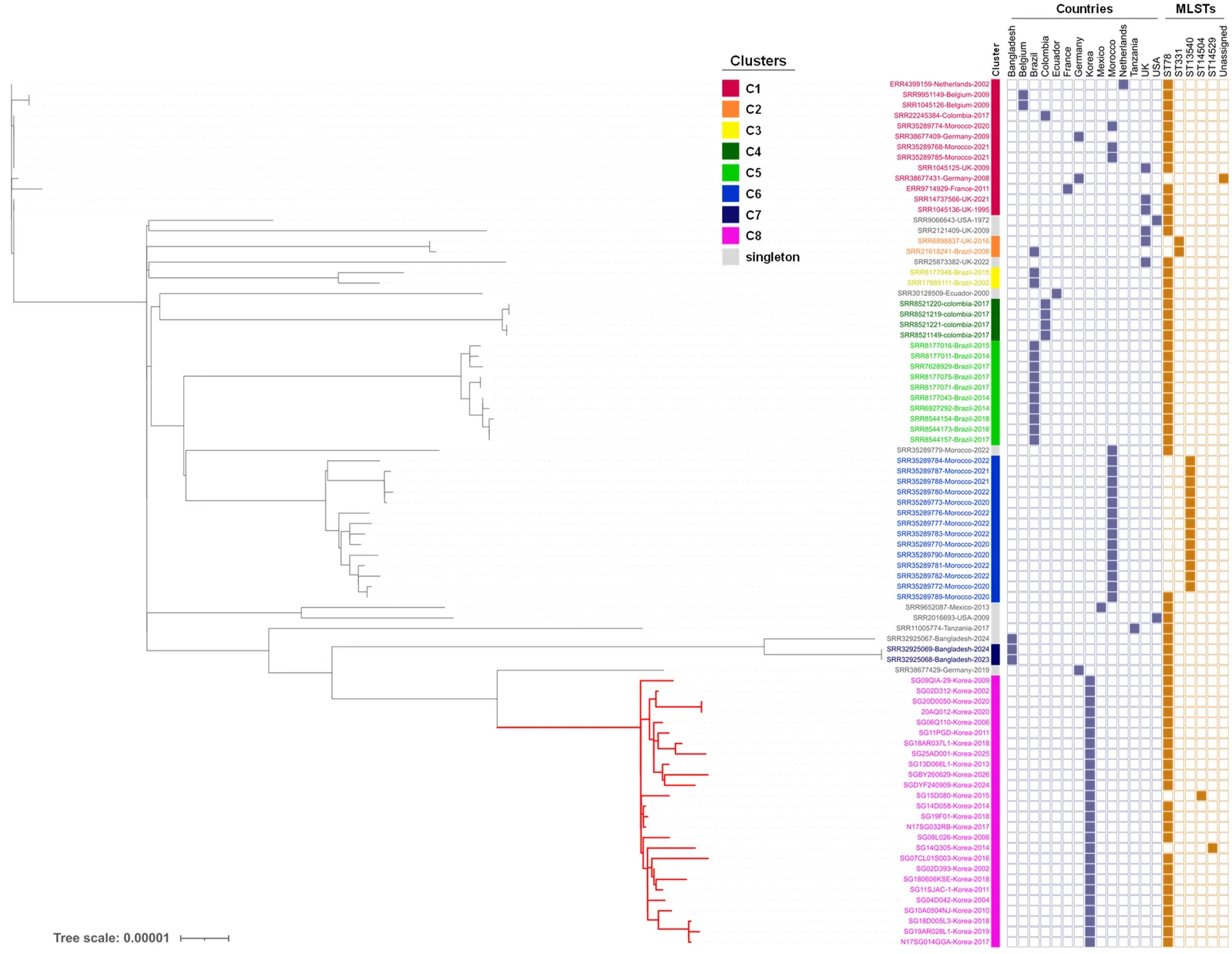
Maximum-likelihood phylogenetic tree based on core-genome SNP alignments for 83 SG isolates. The clusters are indicated by the colors of the tip labels, and the heatmap represents cluster assignments, countries of origin, and multi-locus sequence types. Branches corresponding to the 26 Korean isolates analyzed in this study are highlighted in red.

The core-genome SNP (cgSNP)-based phylogenetic relationships and STs of the 83 SG isolates are presented in Figure 1. At a cutoff value of 100 cgSNPs, the isolates were classified into eight clusters and 10 singletons. All clusters except for C1 and C2 comprised isolates from a single country. Notably, all 26 Korean isolates were assigned to cluster C8. The other country-specific clusters were C3 (Brazil), C4 (Colombia), C5 (Brazil), C6 (Morocco), and C7 (Bangladesh). Cluster C1 comprised strain SG9 (SRR1045136), the 9R-derived vaccine strains SG9R (SRR1045125) and 9R-Col (SRR22245384), and isolates from various countries, whereas Cluster C2 comprised isolates from Brazil and the UK. No distinct clustering patterns were observed according to collection year or host. The human-derived strain SRR9066643 and the equine-derived strain SRR9652087 were each identified as singletons.

The maximum pairwise cgSNP distances within clusters ranged from 0 to 62 SNPs, and the maximum pairwise distance among the Korean isolates was 62 SNPs (Tables S3 and S4). The minimum cgSNP distance between Cluster C8 and isolates outside the cluster was 145 SNPs.

Additionally, five STs (ST78, ST331, ST13540, ST14504, and ST14529) were identified among the 83 isolates. ST78 was the predominant ST among all isolates, including the Korean isolates. ST331 was identified in one isolate each from Brazil and the UK, ST13540 in 13 isolates from Morocco, and ST14504 and ST14529 in one Korean isolate each. One isolate from Germany contained an unassigned ST.

### 3.3. Bayesian Phylogenetic Analysis

**Figure 2:**
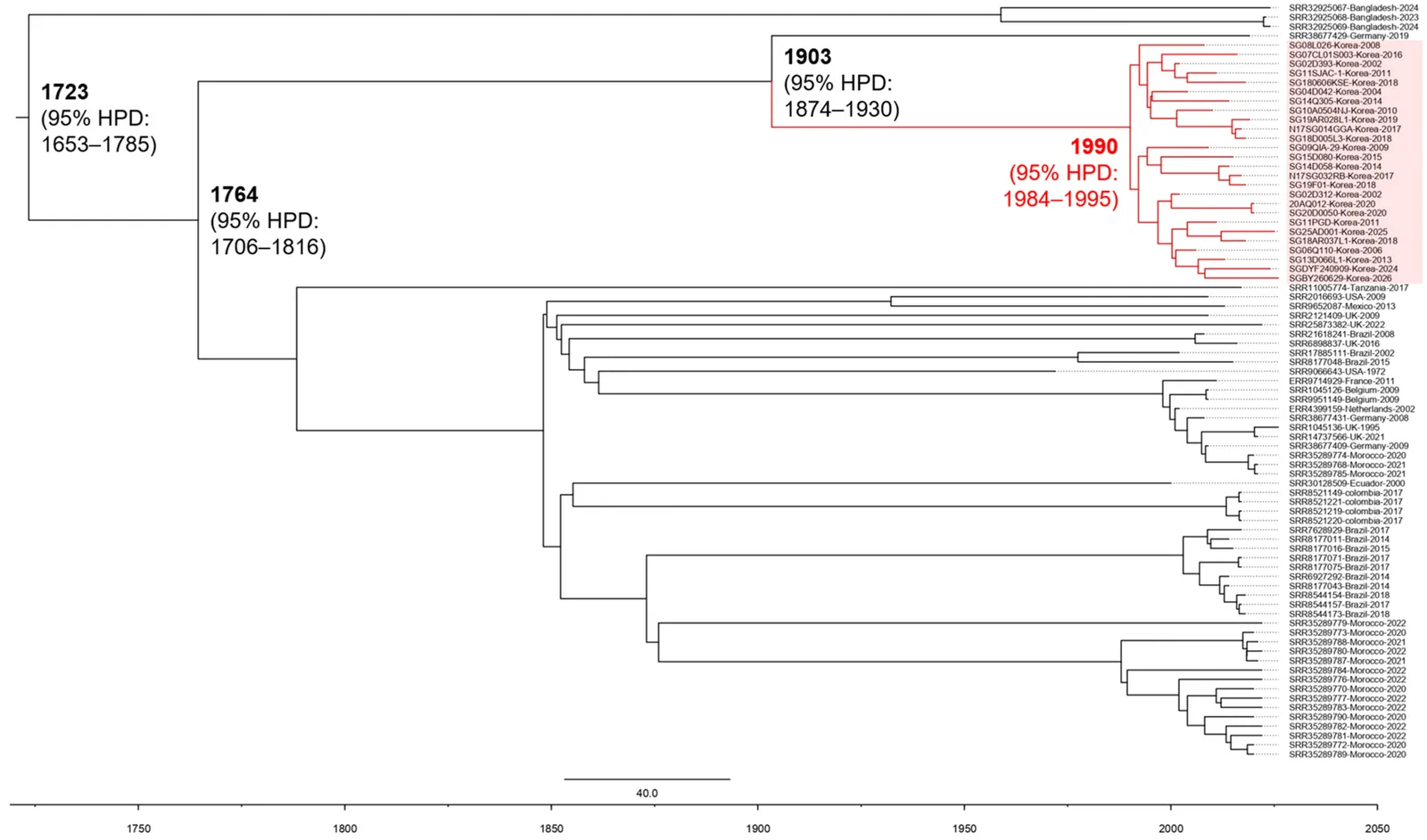
Bayesian maximum clade credibility tree based on core-genome SNP alignments for 81 SG isolates. Branches corresponding to the 26 Korean isolates analyzed in this study are highlighted in red, with the time scale shown along the X-axis.

The time-scaled Bayesian phylogeny of the 81 SG isolates, excluding two vaccine strains (SRR1045125_SG9R and SRR22245384_9R_Col), constructed to investigate their evolutionary history and temporal relationships, is presented in Figure 2. The estimated mean substitution rate was 3.0609 × 10^−4^ (95% HPD: 2.429 × 10^−4^–3.716 × 10^−4^) substitutions per site per year. The time to the most recent common ancestor (tMRCA) of the 81 SG isolates was estimated to be approximately 1723 (95% HPD: 1653–1785), followed by the divergence of the Bangladeshi lineage from the remaining isolates. The tMRCA of the two major lineages—one comprising the Korean isolates and a German isolate (SRR38677429) and the other comprising the remaining global isolates—was estimated to be approximately 1764 (95% HPD: 1707–1816). The Korean lineage and German isolate (SRR38677429) demonstrated a tMRCA of 1903 (95% HPD: 1874–1930), whereas the 26 Korean isolates demonstrated a tMRCA of 1990 (95% HPD: 1984–1995).

### 3.4. SPIs and Virulence Genes

**Figure 3:**
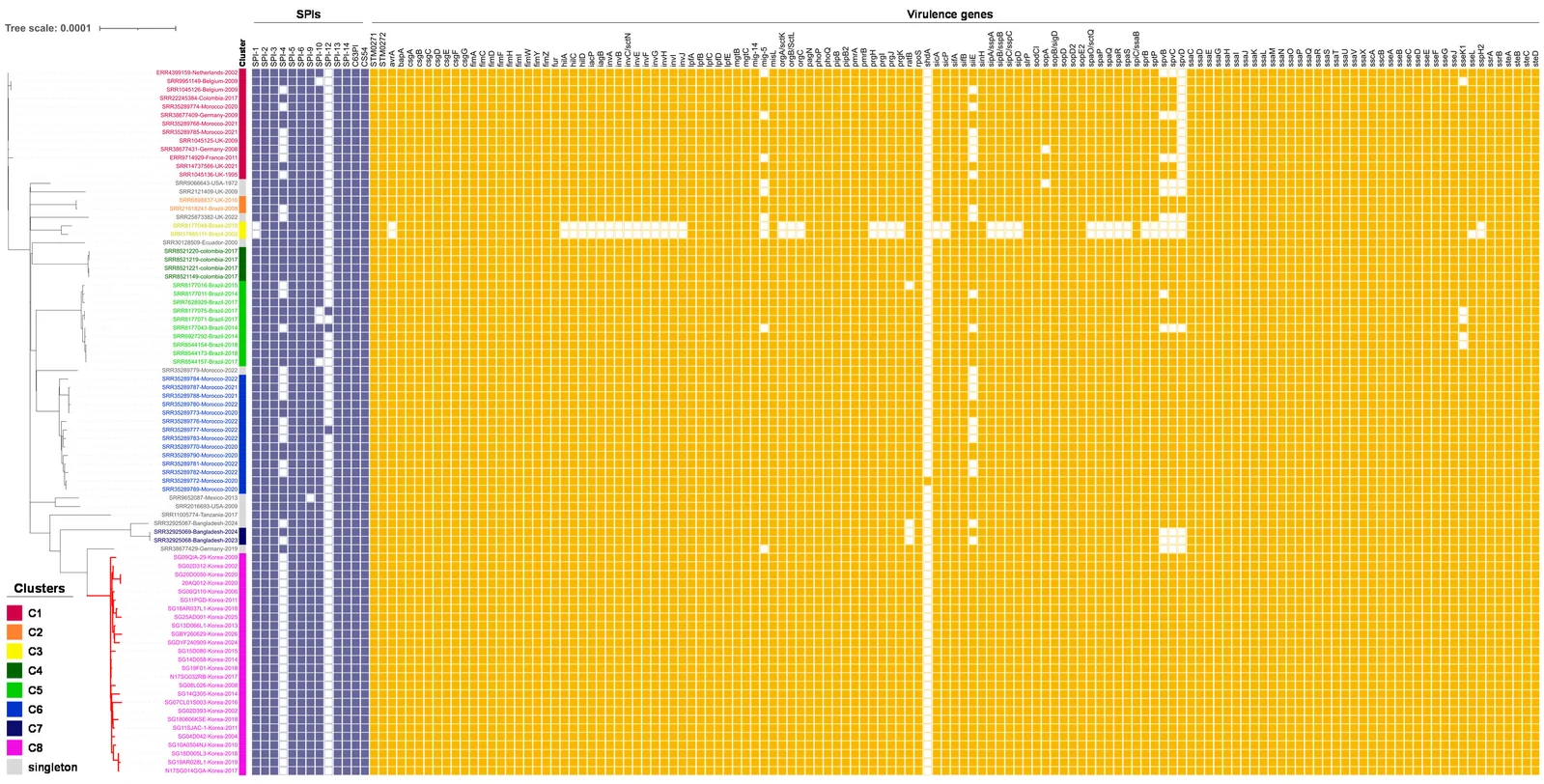
Distribution of *Salmonella* pathogenicity islands and virulence genes among 83 SG isolates in relation to the core-genome SNP phylogeny. The clusters are indicated by the colors of the tip labels, and the respective categories are represented above the heatmap. Branches corresponding to the 26 Korean isolates analyzed in this study are highlighted in red.

The cgSNP phylogeny showing mapped SPI and virulence gene profiles of the 83 SG isolates is presented in Figure 3. Eight pathogenicity islands (SPI-2, SPI-3, SPI-5, SPI-6, SPI-13, SPI-14, C63PI, and CS54) were detected in all isolates. SPI-9, SPI-1, SPI-10, SPI-4, and SPI-12 were detected in 82 (98.8%), 81 (97.6%), 79 (95.2%), 34 (41.0%), and three (3.6%) isolates, respectively.

A total of 129 virulence genes were identified among the 83 isolates, with individual isolates harboring 87–129 genes and showing variation in their virulence gene profiles. Notably, two isolates, SRR8177048 and SRR17885111, in which SPI-1 was not detected, lacked SPI-1 Type III secretion system (T3SS)-associated virulence genes, including *hilA*, *hilC*, *hilD*, *invA*–*C*, *invE*–*J*, *orgA*–*C*, *prgH*–*K*, *sicA*, *sipA*–*D*, *spaO*–*S*, and *sptP*. In addition, the absence of one or more of the *mig-5*, *siiE*, *sopA*, *spvB*, *spvC*, *spvD*, and *sseK1* genes was observed in several isolates in Cluster C1, which included the vaccine strains. The absence of some of these genes, as well as *ratB*, *sseL*, and *sspH2*, was also observed in isolates from other clusters. The *shdA* gene was detected only in one isolate.

### 3.5. Resistance Determinants and Plasmid Replicons

**Figure 4:**
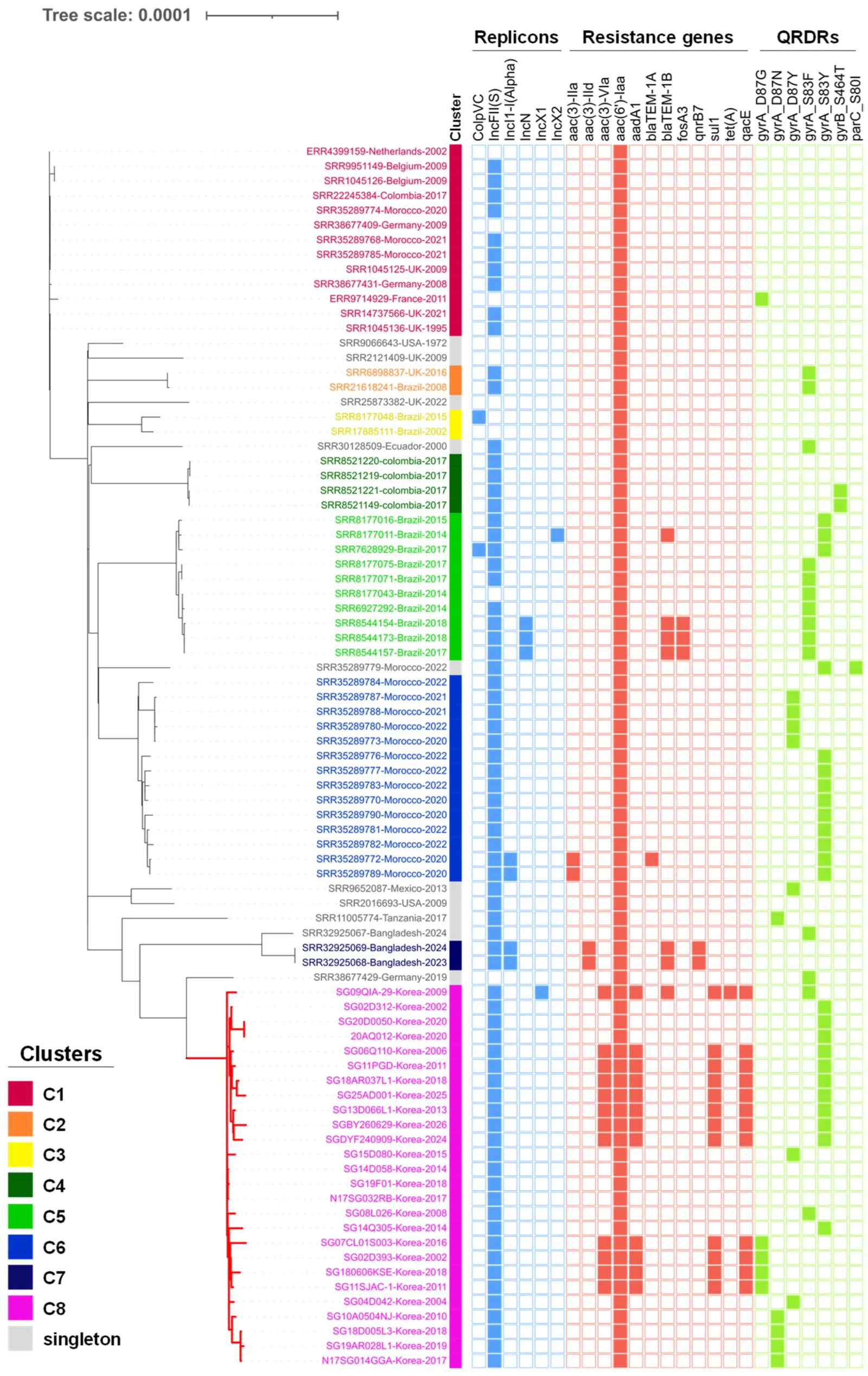
Distribution of plasmid replicons, antimicrobial and disinfectant resistance genes, and point mutations in quinolone resistance-determining region among 83 SG isolates in relation to the core-genome SNP phylogeny. The clusters are indicated by the colors of the tip labels, and the respective categories are represented above the heatmap. Branches corresponding to the 26 Korean isolates analyzed in this study are highlighted in red.

The cgSNP phylogeny showing the mapped plasmid replicons, antimicrobial and disinfectant resistance genes, and quinolone resistance-associated point mutations of the 83 SG isolates is presented in Figure 4. Six plasmid replicon types were identified, with IncFII(S) being most prevalent and detected in 73 isolates. The other plasmid replicons were generally detected in combination with IncFII(S): IncI1-I(Alpha) in four isolates (4.8%), IncN in three (3.6%), ColpVC in two (2.4%), and IncX1 and IncX2 in one isolate each (1.2%). Of these, ColpVC was detected alone in one isolate.

Eleven AMR genes and one disinfectant resistance gene were identified. The *aac(6′)-Iaa* gene was detected in all isolates. In particular, *aac(3)-IIa*, *aac(3)-IId*, *bla*_TEM-1A_, *bla*_TEM-1B_, *fosA3*, and *qnrB7* were detected in isolates harboring IncI1-I(Alpha), IncN, or IncX2 replicons. A resistance gene profile comprising the *aac(3)-VIa*, *aadA1*, *sul1*, and *qacEΔ1* genes was observed in 12 Korean isolates. Of these, 11 isolates, excluding SG09QIA-29, harbored only the IncFII(S) plasmid replicon, with no other plasmid replicon types detected. The IncX1 replicon was also detected in SG09QIA-29, as well as the *bla*_TEM-1B_ and *tet(A)* genes.

Furthermore, seven point mutations in the quinolone resistance-determining region (QRDR) were identified, including five in *gyrA* (S83F, S83Y, D87G, D87N, and D87Y), one in *gyrB* (S464T), and one in *parC* (S80I). Most isolates harbored one or two QRDR mutations; however, no point mutations were detected in Clusters C3 and C7, whereas only one isolate in Cluster C1 harbored a QRDR mutation.

## 4. Discussion

Despite long-standing efforts to control FT, SG continues to affect poultry populations in many parts of the world, with cases reported across multiple continents [3]. In South Korea, FT has occurred sporadically in recent years rather than showing an endemic pattern; nevertheless, the continued detection of SG raises important questions regarding its circulation and genetic characteristics. Therefore, this study comprehensively analyzed 26 SG isolates obtained from poultry in South Korea between 2002 and 2026, together with 57 publicly available SG genomes from diverse geographical regions.

Previous studies have reported a phylogeographic structure in SG, with genetically related isolates tending to cluster according to geographical origin [12,13]. A similar geographical pattern was observed in this study, with six of the eight clusters, excluding Clusters C1 and C2, comprising isolates from a single country. This geographical clustering suggests that SG populations may circulate predominantly within individual countries, with relatively limited genetic exchange between geographically separated populations. However, Cluster C2 contained isolates from both the UK and Brazil, indicating that spread across countries may also occur.

All Korean isolates were also assigned to Cluster C8, with a maximum pairwise cgSNP distance of 62 SNPs within the cluster. Given that Korean isolates were collected over a 25-year period from 2002 to 2026, the genetic variation observed within Cluster C8 may reflect the accumulation of genomic variation during the long-term circulation and diversification of SG in South Korea. However, the minimum cgSNP distance between Cluster C8 and non-C8 isolates was 145 SNPs, more than twice the maximum distance observed within Cluster C8. This clear genetic separation indicates that, despite the genetic diversity observed among Korean isolates, they formed a genetically related group distinct from SG populations from other geographical regions.

Interestingly, Cluster C1 represented a notable exception to the geographical pattern, comprising SG9 (SRR1045136) and the 9R-derived vaccine strains SG9R (SRR1045125) and 9R-Col (SRR22245384). This cluster also included MB4523 (SRR1045126), a field strain isolated during a FT outbreak in Belgium that was reported to be genetically closely related to the SG9R vaccine strain [10]. The SG9R live vaccine has been widely used to control FT, and distinguishing vaccine-related strains from circulating field strains is essential for epidemiological investigations and tracing the sources and transmission of FT outbreaks [37]. Moreover, several researchers have raised the possibility that isolates derived from the SG9R vaccine strain may revert to a more virulent phenotype [10,38].

In South Korea, Korean SG isolates identified as SG9R by a differential multiplex PCR system exhibited characteristics distinct from those of the typical SG9R vaccine strain and have been described as SG9R-like [6,20]. Because the present study focused on the genomic characteristics of circulating field strains of SG in South Korea, isolates identified as SG9R by differential multiplex PCR were excluded from the analysis. This may explain the absence of Korean isolates in the SG9R-related Cluster C1. Nevertheless, the emergence of SG9R-like field strains in South Korea warrants further genomic investigation of SG9R and SG9R-like isolates to clarify their genetic and epidemiological relationships with vaccine-derived strains.

Five sequence types (ST78, ST331, ST13540, ST14504, and ST14529) were identified among the 83 SG isolates, along with one unassigned ST profile. ST78 has been frequently reported in SG isolates [12,13] and was also predominant among Korean isolates. These observations suggest that ST78 is widely distributed across geographically diverse SG populations. Although ST331 has been reported in SG [12,13], the identification of ST13540, ST14504, ST14529, and an unassigned ST in SG has not been previously reported, indicating greater ST diversity than that previously recognized. Notably, different STs within the same *Salmonella* serovar may exhibit distinct pathogenicity; for example, *Salmonella Typhimurium* ST313 has been associated with a more invasive phenotype than the commonly identified ST19 [39]. The identification of previously unreported STs in Korean isolates, such as ST14504 and ST14529, highlights the need for continued monitoring, as these STs may exhibit distinct pathogenicity.

The Bayesian phylogenetic analysis provided further insight into the evolutionary history and temporal diversification of SG. The tMRCA of the 81 SG isolates, excluding the two vaccine strains, was estimated to be approximately 1723. This estimate predates the first recognition of FT in 1889 [40], suggesting that the ancestral SG lineage may have existed for an extended period before FT was formally recognized. Following the early divergence of the Bangladeshi lineage, a subsequent divergence around 1764 separated the lineage comprising the Korean isolates and a German isolate from the broader global lineage containing isolates from South America, North America, Europe, and Africa. This evolutionary divergence further supports the genetic distinction observed between the Korean isolates and isolates from the other countries in the phylogenetic tree.

The Korean isolates and the German isolate showed a more recent tMRCA of 1903, followed by 1990 for the 26 Korean isolates. Notably, the tMRCA of the Korean isolates was temporally close to the first reported field case of FT from South Korea in 1992. This temporal proximity may suggest a temporal association between the estimated diversification of the Korean SG lineage and the first reported FT case in South Korea in 1992. However, because temporal proximity alone cannot establish the timing or geographical origin of SG introduction into South Korea, further analyses incorporating a larger number of historical SG isolates from South Korea, along with additional isolates from other countries representing a broader geographical and temporal range, would be necessary to better clarify the evolutionary relationships of the Korean SG lineage and the timing of its diversification.

SPIs are genomic regions that harbor virulence-associated genes involved in *Salmonella* infection, including host-cell invasion and intracellular survival [41]. The roles of several SPIs in SG infection have been investigated in chickens [42,43,44]. Jones et al. [42] reported that disruption of the SPI-1 T3SS reduced the ability of SG to invade avian cells but did not compromise its virulence in chickens, whereas disruption of the SPI-2 T3SS impaired survival within chicken macrophages and attenuated virulence in chickens. Similarly, Hu et al. [43] demonstrated that deletion of two genes located within SPI-14 significantly attenuated SG virulence in chickens, resulting in lower bacterial loads in the liver and spleen and no mortality compared with the wild-type strain.

Consistent with the reported importance of SPI-2 and SPI-14 in SG virulence, both SPIs were detected in all 83 isolates analyzed in this study. In contrast, SPI-1 was detected in 81 isolates but was absent in two isolates, which also lacked multiple SPI-1 T3SS-associated virulence genes. Given that previous studies have demonstrated that disruption of the SPI-1 T3SS does not necessarily compromise SG virulence in chickens [42], the absence of SPI-1 in SG may have different implications for virulence than the absence of other SPIs, such as SPI-2 and SPI-14. However, the potential effects of these genomic differences on SG virulence remain to be clearly determined. Moreover, differences in the distribution of other SPIs were also observed among the SG isolates, but no clear associations with country or collection year were identified. Because the roles of many SPIs in SG remain incompletely characterized, further characterization of these SPIs is needed to better understand the differences in SPI profiles observed in this study.

In addition, differences in virulence gene profiles were observed among SG isolates. Notably, the absence of several virulence genes was more frequently identified in Cluster C1, which included vaccine strains, and their absence may be associated with attenuation [13,45,46]. However, despite the attenuation of SG9R, some virulence genes may remain intact or harbor mutations that may contribute to residual pathogenicity [10,45]. The absence of some virulence genes was observed not only in Cluster C1 but also in isolates from other clusters. These variations in virulence gene profiles highlight the importance of elucidating the contributions of individual genes to SG pathogenicity, and further experimental validation, including virulence assays, is needed to determine whether the observed genomic differences are associated with differences in SG pathogenicity.

Moreover, recent studies have employed targeted gene deletion as a genetic attenuation strategy for developing SG vaccines [47,48]. Therefore, the characterization of virulence gene variations in SG performed in this study may provide valuable information for identifying candidate genes for further functional evaluation in the development of novel attenuated SG vaccines, while considering the potential contributions of retained or mutated virulence genes to residual pathogenicity.

Plasmids serve as important vehicles for maintaining and disseminating genes associated with virulence and antimicrobial and disinfectant resistance in *Salmonella* [49,50]. In this study, six plasmid replicon types were identified, with IncFII(S) being the most prevalent, detected in 73 of 83 SG isolates. Previous studies have reported an association between the IncFII(S) replicon and the *spv* virulence operon in *Salmonella*. The IncFII(S) replicon was present in most plasmids harboring the *spv* operon in *Salmonella Typhimurium*, I 4,[5],12:i:-, Dublin, and Enteritidis [51] and was also reported to co-occur with *spvB*–*D* in *Salmonella Enteritidis* [52]. Consistent with these observations, *spvB*–*D* was absent from all isolates in which the IncFII(S) replicon was not detected (Table S2), suggesting that this association is also present in SG.

Additional plasmid replicons, including IncI1-I(Alpha), IncN, ColpVC, IncX1, and IncX2, were also identified. Several AMR genes, including *aac(3)-IIa*, *aac(3)-IId*, *bla*_TEM-1A_, *bla*_TEM-1B_, *fosA3*, and *qnrB7*, were detected in isolates carrying these plasmid replicons, except for ColpVC. IncI1, IncN, and IncX plasmid replicons have been associated with the carriage and dissemination of AMR genes in *Salmonella* [1]. Therefore, the observed co-occurrence of these plasmid replicons and AMR genes is consistent with their potential involvement in the dissemination of plasmids carrying AMR determinants.

Interestingly, a resistance gene profile comprising AMR (*aac(3)-VIa*, *aadA1*, and *sul1*) and disinfectant (*qacEΔ1*) genes was observed in 12 Korean isolates. Eleven of these isolates harbored only the IncFII(S) plasmid replicon, whereas SG09QIA-29 also harbored IncX1, together with *bla*_TEM-1B_ and *tet(A)*. These findings suggest that IncFII(S) plasmids may have accumulated diverse resistance determinants through recombination and mobile genetic element-mediated events [53,54]. Moreover, the acquisition of additional plasmids, such as IncX1, may further contribute to the accumulation of a broader range of resistance determinants in SG.

Phenotypic antimicrobial susceptibility data from the 26 Korean isolates provided additional context for these genotypic findings (Table S5). Resistance to gentamicin and sulfisoxazole was observed in 12 isolates each, with 11 isolates in each group harboring the corresponding resistance genes, *aac(3)-VIa* and *sul1*, respectively. The single ampicillin-resistant isolate harbored *bla*_TEM-1B_, while the tetracycline-resistant isolate carried *tet(A)*. Streptomycin resistance was observed in 21 isolates, and among the 12 isolates carrying *aadA1*, 11 exhibited high MIC values (>64 µg/mL). These findings indicate that the detected AMR genes were generally consistent with the corresponding phenotypic resistance profiles. In particular, the association between *aadA1* carriage and elevated streptomycin MICs suggests that this gene may contribute to high-level streptomycin resistance. This highlights the importance of considering MIC values alongside AMR gene profiles when evaluating antimicrobial resistance in SG.

The chromosomally encoded *aac(6′)-Iaa* gene, which is commonly found in *Salmonella*, was detected in all SG isolates [55]. However, phenotypic resistance to aminoglycosides varied among the 26 Korean isolates, indicating that the presence of this gene alone does not necessarily confer resistance. Although this chromosomally encoded gene may remain cryptic without conferring phenotypic resistance under normal conditions, a previous study demonstrated that transposon-mediated activation of *aac(6′)-Iaa* can confer aminoglycoside resistance [56]. Moreover, streptomycin resistance was also observed in nine isolates lacking *aadA1*, suggesting the possible involvement of additional resistance determinants or mechanisms not identified in the present analysis. Therefore, further investigations into resistance-associated genes, chromosomal mutations, and gene expression are warranted to elucidate the molecular mechanisms underlying these genotype–phenotype discrepancies and the potential activation of cryptic resistance determinants.

QRDR mutations are frequently observed in *Salmonella* and are associated with quinolone resistance [54]. In this study, seven QRDR mutations were identified, including five in *gyrA* (S83F, S83Y, D87G, D87N, and D87Y), one in *gyrB* (S464T), and one in *parC* (S80I). No QRDR mutations were detected in Clusters C3 and C7, and only one isolate in Cluster C1, which was associated with vaccine strains, harbored a mutation. Given that QRDR mutations are generally selected under quinolone exposure [57], their limited occurrence in these clusters may reflect differences in quinolone selective pressure among the respective SG populations. Notably, QRDR mutations were detected in all but three Korean isolates. This high prevalence may be associated with the widespread use of fluoroquinolones for the prevention and treatment of FT in the Korean poultry industry [58].

Consistent with these genotypic findings, phenotypic resistance to nalidixic acid was observed in 23 of the 26 Korean isolates, all of which harbored *gyrA* mutations (Table S5). These findings support previous reports associating *gyrA* mutations with quinolone resistance [59]. In contrast, only 17 of the 23 isolates carrying *gyrA* mutations exhibited phenotypic resistance to ciprofloxacin. Notably, ciprofloxacin resistance patterns differed according to the type of *gyrA* mutation. All isolates harboring S83Y, S83F, or D87Y substitutions were classified as ciprofloxacin resistant. In contrast, only one of the four isolates carrying D87G and one of the four isolates carrying D87N were classified as ciprofloxacin resistant. These differences suggest that ciprofloxacin resistance phenotypes may vary depending on the specific amino acid substitution in *gyrA*. Further investigations are therefore warranted to clarify the relative contributions of individual QRDR mutations to fluoroquinolone resistance in SG.

Collectively, the SG isolates exhibited diverse genetic determinants associated with antimicrobial and disinfectant resistance, with phenotypic antimicrobial susceptibility data from the 26 Korean isolates generally supporting the corresponding genotypic findings. These results highlight the value of combining WGS-based resistance gene profiling with phenotypic susceptibility testing to better understand antimicrobial resistance characteristics in SG. In particular, the genotypic resistance profiles observed among the Korean isolates highlight the importance of prudent antimicrobial use and appropriate disinfection practices to improve FT control and limit the emergence and spread of resistant bacteria. However, phenotypic antimicrobial susceptibility testing was performed only on the 26 Korean isolates, limiting the assessment of genotype–phenotype relationships across the entire dataset. Therefore, the observed associations between AMR determinants and phenotypic resistance should be interpreted with caution, as these findings may not fully reflect the resistance characteristics of SG isolates from other countries.

Several limitations should be considered when interpreting the findings of this study. Although the 26 Korean isolates were collected across different years and poultry production types, the relatively small sample size over the extended collection period from 2002 to 2026, together with the predominance of isolates from laying hens, suggests that these isolates may not fully represent the overall SG population in South Korea. Furthermore, the limited geographical information on the Korean isolates precluded a detailed assessment of regional differences in their genetic characteristics. Publicly available SG whole-genome sequence data from different countries remain limited, particularly in terms of geographical coverage. In addition, differences in disease surveillance and diagnostic practices among countries may have influenced the collection and availability of SG isolates, potentially introducing additional sampling bias into comparative genomic analysis. Consequently, the uneven temporal and geographical distribution of the isolates, along with differences in surveillance and diagnostic practices among countries, may limit conclusions regarding population structure and transmission pathways within and between countries. Nevertheless, our findings provide valuable baseline genomic information on the genetic characteristics and phylogenetic relationships of SG isolates from South Korea and other countries. These findings also highlight the need for greater availability and sharing of WGS data from SG isolates collected across more diverse countries and time periods to enable more comprehensive and precise WGS-based analyses.

## 5. Conclusions

In summary, to the best of our knowledge, this is the first study to comprehensively characterize SG isolates from poultry in South Korea using WGS and compare their genomic characteristics with those of SG isolates from other countries. The Korean SG isolates showed distinct phylogenetic and epidemiological characteristics compared with isolates from other countries. Moreover, genomic analyses revealed diverse genetic determinants associated with virulence and antimicrobial and disinfectant resistance among the Korean SG isolates. WGS data from South Korea and other countries are limited; therefore, the SG isolates included in this study may not fully represent the genomic diversity of SG. Nevertheless, our findings provide valuable genomic and epidemiological insights into SG and establish a foundation for future WGS-based studies involving larger and more geographically diverse isolate collections, as well as for improved surveillance and control of FT.

## Data Availability

Data are available in a publicly accessible repository. The WGS data have been deposited in the NCBI SRA under BioProject accession number PRJNA1533643, with BioSample accession numbers SAMN63375196–SAMN63375221.

## Supplementary Materials

The following supporting information can be downloaded at https://www.mdpi.com/article/10.3390/ani16213404/s1, Table S1: Information on the 83 SG isolates analyzed in this study; Table S2: Profiles of MLST, SPIs, plasmid replicons, virulence genes, antimicrobial resistance genes, and QRDR mutations in the 83 SG isolates analyzed in this study; Table S3: Pairwise SNP distance matrix of the 83 SG isolates analyzed in this study; Table S4: Characteristics of SNP distance-based clusters among the 83 SG isolates analyzed in this study; Table S5: Antimicrobial resistance gene profiles and minimum inhibitory concentration values of the 26 Korean SG isolates analyzed in this study.

## Abbreviations

The following abbreviations are used in this manuscript:

FT	fowl typhoid
SG	*Salmonella enterica* subsp. *enterica* serovar Gallinarum biovar Gallinarum
AMR	antimicrobial resistance
WGS	whole-genome sequencing
PCR	polymerase chain reaction
ST	sequence type
SPI	*Salmonella* pathogenicity island
SNP	single-nucleotide polymorphism
cgSNP	core-genome single-nucleotide polymorphism
tMRCA	time to the most recent common ancestor
T3SS	Type III secretion system
QRDR	quinolone resistance-determining region
